# RocA Binds CsrS To Modulate CsrRS-Mediated Gene Regulation in Group A *Streptococcus*

**DOI:** 10.1128/mBio.01495-19

**Published:** 2019-07-16

**Authors:** Nicola N. Lynskey, Jorge J. Velarde, Meredith B. Finn, Simon L. Dove, Michael R. Wessels

**Affiliations:** a Division of Infectious Diseases, Boston Children’s Hospital, Boston, Massachusetts, USA; b Department of Pediatrics, Harvard Medical School, Boston, Massachusetts, USA; University of Mississippi Medical Center; St. Jude's Children's Research Hospital; Univ. of Texas Houston Medical School

**Keywords:** *Streptococcus pyogenes*, gene regulation, pathogenesis, regulatory proteins, virulence

## Abstract

The orphan regulator RocA plays a critical role in the colonization and pathogenesis of the obligate human pathogen group A *Streptococcus*. Despite multiple lines of evidence supporting a role for RocA as an auxiliary regulator of the control of virulence two-component regulatory system CsrRS (or CovRS), the mechanism of action of RocA remains unknown. Using a combination of *in vitro* and *in vivo* techniques, we now find that RocA interacts with CsrS in the streptococcal membrane via its N-terminal region, which contains seven transmembrane domains. This interaction is essential for RocA-mediated regulation of CsrRS function. Furthermore, we demonstrate that RocA forms homodimers via its cytoplasmic domain. The serotype-specific RocA truncation in M3 isolates alters this homotypic interaction, resulting in protein aggregation and impairment of RocA-mediated regulation. Taken together, our findings provide insight into the molecular requirements for functional interaction of RocA with CsrS to modulate CsrRS-mediated gene regulation.

## Abstract (precis)

Bacterial two-component regulatory systems, comprising a membrane-bound sensor kinase and cytosolic response regulator, are critical in coordinating the bacterial response to changing environmental conditions. More recently, auxiliary regulators which act to modulate the activity of two-component systems, allowing integration of multiple signals and fine-tuning of bacterial responses, have been identified. RocA is a regulatory protein encoded by all serotypes of the important human pathogen group A *Streptococcus*. Although RocA is known to exert its regulatory activity via the streptococcal two-component regulatory system CsrRS, the mechanism by which it functions was unknown. Based on new experimental evidence, we propose a model whereby RocA interacts with CsrS in the streptococcal cell membrane to enhance CsrS autokinase activity and subsequent phosphotransfer to the response regulator CsrR, which mediates transcriptional repression of target genes.

## INTRODUCTION

Regulation of virulence factor production is integral to bacterial survival and pathogenesis. A major mechanism by which bacteria detect and respond to environmental cues involves the integration of signals from two-component regulatory systems (TCS) (1). While many variations exist, these systems typically comprise a membrane-bound sensor kinase and a cognate cytoplasmic response regulator, the activity of which is modulated by phosphorylation (1). Environmental signals stimulate or inhibit auto-kinase activity of the sensor and downstream phosphorylation of the response regulator.

Thirteen TCS have been identified in the important human pathogen group A *Streptococcus* (Streptococcus pyogenes or GAS). Among these, CsrRS (also known as CovRS), has been shown to play a critical role in streptococcal colonization and disease pathogenesis by regulating the expression of ∼10% of the GAS genome, including genes encoding several key virulence factors (2–6). Naturally occurring loss-of-function mutations in CsrRS have been identified in up to 40% of invasive disease isolates (7–9). The increased virulence of such strains has been attributed to derepression of transcription of CsrRS-regulated virulence factors, such as the hyaluronic acid capsule and the cotranscribed toxins NAD^+^ glycohydrolase (NADase) and streptolysin O (4).

Two environmental signals have been shown to regulate expression of genes in the CsrRS regulon: extracellular magnesium concentration and the human antimicrobial peptide LL-37 (6, 10–12). Exposure of GAS to elevated concentrations of Mg^2+^ stimulates phosphorylation of CsrR in a CsrS-dependent manner and results in repression of most CsrRS-regulated genes (6, 13). LL-37 has been shown to bind to CsrS *in vitro* (14), and exposure of GAS to subinhibitory concentrations of LL-37 results in reduced phosphorylation of CsrR and increased expression of the same genes that are repressed by Mg^2+^ (11, 12, 15).

Auxiliary regulators that interact with TCS to form multicomponent signal transduction systems have been identified in several bacterial species (16–21). These proteins integrate diverse inputs to modulate TCS by direct protein-protein interactions, which ultimately dictate the phosphorylation state of the response regulator and subsequent transcriptional regulation of target genes (19). Auxiliary proteins can act in a variety of ways on either the sensor kinase or the response regulator. Different classes of auxiliary regulators include accessory phosphatases, such as the Escherichia coli protein CheZ, which dephosphorylates the response regulator CheY (20), and antikinases, such as the Bacillus subtilis protein KipI, which inhibits the kinase activity of the histidine kinase KinA via the catalytic domain (21). Other membrane-bound regulators have been shown to interact with the transmembrane domains (TMDs) of histidine kinases to modulate either kinase or phosphatase activity, examples of which include the group B streptococcal protein Abx1, which impedes the kinase activity of CsrS (16).

The orphan regulator RocA was originally identified as a positive regulator of the CsrRS TCS (22). More recent studies suggest that RocA acts as an auxiliary regulator of CsrRS, a hypothesis supported by transcriptome sequencing (RNA-seq) experiments showing the RocA regulon to be entirely included within that of CsrRS (23–25). Indeed, naturally occurring mutations in RocA result in derepression of CsrRS-regulated virulence genes and enhanced virulence of the affected strains (23, 25, 26). Such mutations or deletion of RocA results in reduced phosphorylation of CsrR, which, in turn, is thought to relieve the repression of CsrRS-regulated gene expression (23). Mg^2+^ retains its regulatory effect on the CsrRS regulon in a RocA mutant, but LL-37 does not (27). These observations are consistent with a model in which, in a RocA mutant, phosphorylation of CsrR can be increased through the interaction of Mg^2+^ with CsrS. However, since CsrR is maximally dephosphorylated at baseline in a RocA mutant, it cannot be further dephosphorylated through the action of LL-37.

Despite a clear role for RocA in modulating CsrRS-mediated regulation in GAS, conserved, M-type-specific, nonsense mutations in *rocA* have been identified in all tested isolates of two important serotypes, M3 (23, 28) and M18 (26). These mutations have been shown to impair CsrRS-mediated gene regulation, thereby increasing production of the hyaluronic acid capsule and other CsrRS-regulated virulence factors (23, 26–28). The M18-specific truncation occurs very early in the RocA protein (amino acid 29 of 451) (26), and no protein fragment is thought to be expressed. In contrast, truncated M3 RocA protein, which results from a frameshift mutation at codon 410 and a subsequent premature stop codon at position 416, is not functional (23, 28). Complementation of *rocA* of serotype M18 strains (*rocA*_M18_) or *rocA*_M3_ with a single copy of the full-length gene is sufficient to restore CsrRS regulatory control (23, 26, 28). Interestingly, overexpression of RocA_M3_ in a RocA-negative background restored CsrRS regulatory activity, suggesting that multiple copies of this truncated protein are sufficient to overcome its structural or conformational defects in modulating CsrRS activity (27). Until now, the mechanism by which RocA_M3_ activity is impaired has not been described.

To better understand the functional relationship between RocA and CsrRS, we investigated the physical association between these two proteins. RocA is a transmembrane protein predicted to be comprised of seven N-terminal TMDs and a C-terminal cytoplasmic domain (CYT) that includes a putative ATPase motif (22). Experiments using strains expressing defined fragments of RocA revealed that the C terminus was necessary and sufficient for RocA homodimerization. However, the cytoplasmic domain alone was not sufficient to restore RocA modulation of CsrRS regulation in a RocA-deficient GAS strain. Using a combination of coimmunoprecipitation in GAS and Lactococcus lactis, we found evidence for a direct interaction between the membrane-associated N-terminal domain of RocA and CsrS. Our data support a model in which RocA binding to CsrS potentiates CsrS autokinase activity and/or inhibits its phosphatase activity, enhancing downstream phosphorylation of CsrR to regulate virulence gene expression.

## RESULTS

### RocA transmembrane domains are sufficient to modulate phosphorylation of CsrR- and CsrRS-regulated gene expression.

**FIG 1:**
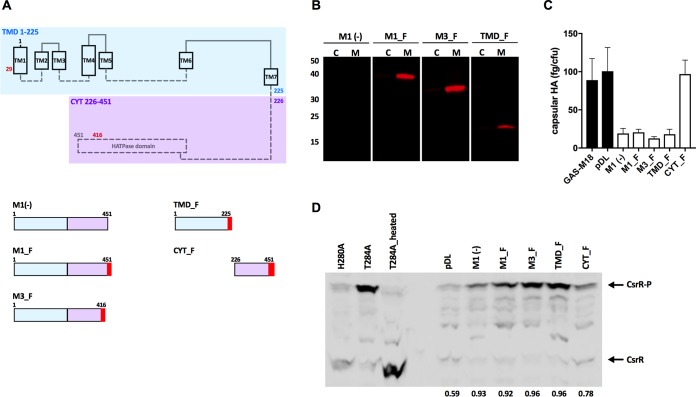
Characterization of GAS strains expressing full-length RocA or RocA fragments. (A) Schematic representation of RocA protein. The region of RocA containing transmembrane domains (TMDs; amino acids 1 to 225) is highlighted in blue, and the cytoplasmic domain (CYT; amino acids 225 to 451) is highlighted in purple. Red numbers denote sites of M3 (amino acid 416)- and M18 (amino acid 29)-specific truncations. RocA fragments expressed in plasmid pDL278 are shown schematically below. HATPase, histidine kinase-like ATPase. (B) Western blot of membrane fractions of GAS M18 strains expressing specific RocA_FLAG_ fragments probed with anti-FLAG antibody. Lanes C, cytosolic fraction; lanes M, membrane fraction. Numbers at the left are molecular masses (in kilodaltons). (C) Quantification of capsular hyaluronic acid of GAS M18 strains expressing RocA_FLAG_ fragments. (D) Western blot for CsrR in GAS lysates separated on 10% Phos-tag polyacrylamide gels for the strains indicated above each lane. CsrR-P denotes phosphorylated CsrR protein. T284A and H280A CsrS mutant GAS strains served as controls (the T284A strain for phosphorylated CsrR and the heated T284A and H280A strains for unphosphorylated CsrR). GAS strains were as follows: GAS M18, the wild-type M18 strain H566; pDL, H566 containing the empty vector pDL278; M1(–), H566 plus pDL_rocA_M1_ (amino acids 1 to 451); M1_F, H566 plus pDL_RocA_M1_FLAG_ (amino acids 1 to 451); M3_F, H566 plus pDL_RocA_M3_FLAG_ (amino acids 1 to 416); TMD_F, H566 plus pDL_RocA_TMD_FLAG_ (amino acids 1 to 225); and CYT_F, H566 plus pDL_RocA_CYT_FLAG_ (amino acids 226 to 451). Numbers beneath the blot indicate the proportions of total CsrR protein that corresponds to the phosphorylated form by image densitometry analysis.

Previous reports identify RocA as a membrane-associated protein based on analysis with protein prediction software (24, 27), which we confirmed using TMPred (29) (Fig. 1A). However, membrane localization of RocA has not been proven *in vivo*. Accordingly, we investigated the subcellular location of RocA. Native expression of RocA within GAS is very low, so to facilitate detection, multiple copies of RocA_M1_ with a C-terminal FLAG tag (RocA_FLAG_) were expressed from plasmid pDL278 in a serotype M18, RocA-negative strain (Fig. 1). The resulting strain was designated M1_F (Table 1). A control strain expressing RocA_M1_ without a FLAG tag [M1(–)] was also generated (Fig. 1B, Table 1). Membrane and cytosolic fractions of strains M1_F and M1(–) were purified, and RocA was detected specifically in the membrane fraction (Fig. 1B). As reported previously, expression of full-length RocA_M1_ in an M18 strain was sufficient to repress capsule expression (26) (Fig. 1C).

**TABLE 1:** Bacterial strains used in this study

Strain	Description	Reference/source
S. pyogenes 854	Wild-type M1	11
S. pyogenes 188	Acapsular mutant of wild-type M3 strain 950771	40
S. pyogenes H566	Wild-type M18	26
L. lactis 1363	Wild type	41
M1(–)	H566 + pDL_rocA_M1_	This study
M1_F	H566 + pDL_rocA_M1_FLAG_	This study
M3_F	H566 + pDL_rocA_M3_FLAG_	This study
TMD_F	H566 + pDL_rocA_TMD_FLAG_	This study
CYT_F	H566 + pDL_rocA_CYT_FLAG_	This study
854_chF	854 expressing *rocA*_FLAG_	This study
854_H280A	854 expressing *csrS*_H280A_	12
854_T284A	854 expressing *csrS*_T284A_	This study
L_RocA_FLAG__CsrS_HIS_	L. lactis + pOri_csrS_HIS__rocA_FLAG_	This study
E. coli KDZif1ΔZ	β-Galactosidase reporter	42

In order to study the separate roles of the RocA transmembrane and cytoplasmic domains, we generated additional plasmids to express separately the N-terminal TMD region and the C-terminal cytoplasmic domain. In addition, we generated a plasmid expressing RocA_M3_ to allow investigation into the activity of this variant of the RocA protein. Sequences encoding these domains were cloned into pDL278 with a C-terminal FLAG tag and transformed into an M18, RocA-negative strain for expression analysis. The resultant GAS strains were designated M3_F (expressing RocA_M3_), TMD_F (expressing RocA amino acids 1 to 225, including all TMDs), and CYT_F (expressing RocA amino acids 226 to 451, corresponding to the cytoplasmic domain of RocA_M1_) (Fig. 1A and B; Table 1). Expression of the N-terminal region, including the TMDs of RocA from the multicopy plasmid pDL278 (strain TMD_F) was sufficient to restore the regulatory activity of RocA, as demonstrated by repression of expression of capsular hyaluronic acid (Fig. 1C), supporting previous reports (27). Regulatory activity appears to require the TMDs, as expression of the cytoplasmic domain alone from the same vector did not restore regulatory control (Fig. 1C).

Previous reports have demonstrated that the regulatory activity of RocA is dependent on the expression of functional CsrS (23), whereby RocA enhances the phosphorylation of the response regulator CsrR, promoting the transcriptional repression of target genes. Based on our observations suggesting that the TMDs of RocA are sufficient for effective modulation of CsrRS-mediated gene regulation, we expected that this fragment of RocA would also be sufficient to modulate the phosphorylation status of CsrR. To test this prediction, we used Phos-tag SDS-PAGE and Western blotting to assess the levels of phosphorylated CsrR (CsrR-P) in cell lysates purified from GAS strains expressing different RocA fragments (Fig. 1D). In this system, Phos-tag reagent incorporated into the SDS-PAGE gel binds to phosphorylated proteins and retards their migration, allowing differentiation between the phosphorylated and unphosphorylated forms of a specific protein by immunoblotting. Using this approach, we found that GAS strains expressing RocA fragments that included the TMDs were associated with increased CsrR phosphorylation compared to that of a strain expressing the RocA cytoplasmic domain or an empty vector control. Additional controls included CsrS mutant GAS strains harboring inactivating mutations in the kinase (H280A) (12) or phosphatase (T284A) domains, which display low and high phosphorylation of CsrR, respectively (30).

Results of these experiments indicate that RocA modulates the phosphorylation status of CsrR and that the TMDs are required for this activity (Fig. 1D). As with the results for capsule expression, the cytoplasmic domain of RocA alone was not sufficient to promote high-level phosphorylation of CsrR. These data strongly implicate RocA as a functional regulator of CsrR phosphorylation.

### RocA interacts with CsrS in the streptococcal membrane.

Our data suggested that only the TMD-containing N-terminal region of RocA is essential for regulatory activity, which has previously been demonstrated to be dependent on the expression of a functional CsrS (23). While multiple lines of evidence support a role for RocA as an auxiliary regulator of CsrRS, a mechanism by which such regulation may occur has not been determined. We hypothesized that RocA and CsrS interact within the streptococcal membrane via the RocA TMDs. To test this hypothesis, we first investigated whether full-length RocA could bind CsrS.

**FIG 2:**
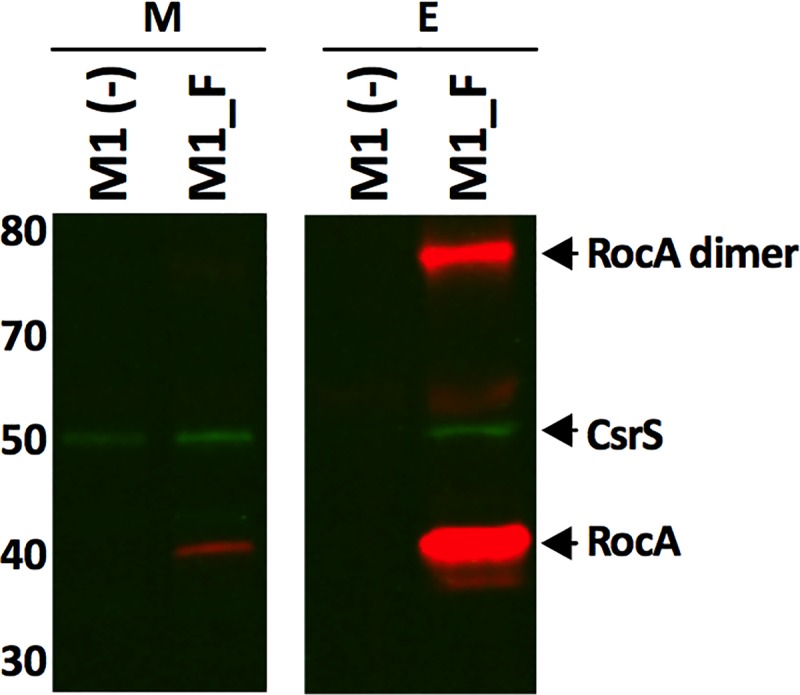
RocA interacts directly with CsrS in the GAS cell membrane. RocA_FLAG_ was expressed from plasmid pDL278 in GAS M18 (M1_F); solubilized membrane preparations (lanes M) were immunoprecipitated using anti-FLAG beads. SDS-PAGE and immunoblotting of the eluted fraction showed enrichment for RocA_FLAG_ and specific coprecipitation of native CsrS in the eluted fraction (lanes E) from strain M1_F but not from the negative control, M1(–), which expresses RocA without the FLAG tag (red, anti-FLAG; green, anti-CsrS).

In order to determine whether RocA forms a complex with CsrS *in vivo*, we performed coimmunoprecipitation experiments for CsrS and RocA with GAS strain M1_F expressing RocA_FLAG_ in the bacterial membrane. For these experiments, GAS membrane fractions were detergent solubilized, and RocA_FLAG_ was purified by affinity chromatography using anti-FLAG beads (Fig. 2). Native CsrS protein coeluted with full-length RocA_FLAG_ from the solubilized membranes of strain M1_F. Importantly, CsrS was not detected in the eluted fraction from the same immunoprecipitation protocol performed with the control strain, M1(–), a result that rules out nonspecific interaction of CsrS with the anti-FLAG beads (Fig. 2). As a further control for the specificity of the binding interaction between RocA and CsrS, a silver-stained gel suggested that RocA_FLAG_ and CsrS accounted for nearly all the protein from strain M1_F eluted from the anti-FLAG affinity resin in the experiment described above (see Fig. S1 in the supplemental material).

10.1128/mBio.01495-19.1 FIG S1 Immunoprecipitation of RocA_FLAG_ from a membrane preparation of GAS strain M1_F pulls down CsrS and only minimal amounts of other proteins. (A) Silver-stained SDS-PAGE gel of the membrane preparations (M) and eluted fractions (E) from the anti-FLAG affinity resin used in the experiments whose results are shown in Fig. 2. The strains tested are M1(–) and M1_F, expressing RocA without and with a FLAG tag, respectively. (B) Anti-FLAG Western blot run on the same samples. The only band observed in the eluted fraction of M1(–) is the nonspecific band representing a protein expressed by GAS that cross-reacts with the anti-FLAG antibody. All the major protein bands observed in the eluted fraction of strain M1_F by silver staining are observed in the anti-FLAG Western blot, suggesting that these bands were specifically pulled down by immunoprecipitation. Many protein bands visible in the membrane preparation of M1(–) by silver staining are not visible in the eluted fraction, in particular, the proteins smaller than 40 kDa. These results suggest that the pulldown is largely free of unrelated proteins, and the coelution of CsrS with RocA_FLAG_ reflects a specific interaction. Download FIG S1, PDF file, 0.5 MB. Copyright © 2019 Lynskey et al. 2019 Lynskey et al. This content is distributed under the terms of the Creative Commons Attribution 4.0 International license.

In order to validate these findings, we expressed both RocA and CsrS in the nonpathogenic Gram-positive bacterium Lactococcus lactis (Table 1). This approach allowed us to overexpress both proteins in a bacterial species in which they play no functional role and are thus tolerated at high concentrations. RocA_FLAG_ and CsrS_His_ were coexpressed from the pOri23 vector. CsrS_His_ was purified from detergent-solubilized membrane preparations using nickel-nitrilotriacetic acid (Ni-NTA) resin. RocA coeluted with CsrS (Fig. S2), suggesting that the two proteins interact within the bacterial membrane.

10.1128/mBio.01495-19.2 FIG S2 RocA interacts directly with CsrS in the cell membrane of a heterologous bacterial species. RocA_FLAG_ and CsrS_His_ were coexpressed from plasmid pOri23 in L. lactis. Bacterial membranes (lane M) were isolated and solubilized, and the proteins were subjected to affinity purification using Ni-NTA resin. SDS-PAGE and immunoblotting of the fraction eluted from the resin (lane E) showed enrichment for CsrS_His_ and coprecipitation of RocA_FLAG_ (red, anti-FLAG; green, anti-His). Download FIG S2, PDF file, 0.1 MB. Copyright © 2019 Lynskey et al. 2019 Lynskey et al. This content is distributed under the terms of the Creative Commons Attribution 4.0 International license.

### RocA TMDs are essential for intramembrane interaction with CsrS.

**FIG 3:**
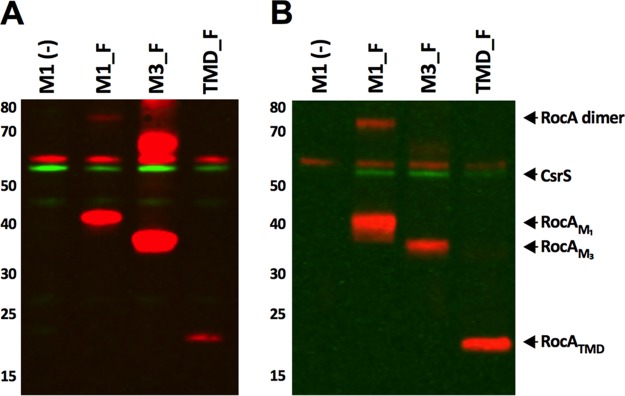
RocA transmembrane domains are sufficient for binding to CsrS. Pulldown experiments of RocA_FLAG_ fragments with CsrS. (A) Western blot of solubilized membrane preparations of GAS M18 expressing RocA_FLAG_ fragments expressed from plasmid pDL278. (B) Western blot of the eluted fraction of an anti-FLAG immunoprecipitation showing enrichment for RocA_FLAG_ fragments of full-length M1, M3 (amino acids 1 to 416), and the transmembrane domain (amino acids 1 to 225). Coprecipitation of CsrS was observed for all RocA_FLAG_ fragments tested (red, anti-FLAG; green, anti-CsrS). See the legend of Fig. 1 for strain designations.

Having demonstrated a specific interaction between RocA and CsrS in the GAS cell membrane, we investigated which domains of the RocA protein were necessary for this interaction. Using the panel of GAS strains expressing different FLAG-tagged RocA fragments (Fig. 1B; Table 1), we performed similar pulldown experiments with detergent-solubilized membrane fractions of strains M3_F and TMD_F (Fig. 3A). RocA_M3_ and RocA_TMD_ FLAG-tagged protein fragments were purified by affinity chromatography, similarly to full-length RocA (Fig. 3B). Following immunoprecipitation of these RocA fragments, native CsrS protein was detected in the eluted fraction with both RocA fragments, unlike in identical experiments performed with control strain M1(–) (Fig. 3B). We therefore conclude that the N-terminal region containing the TMDs of RocA is sufficient to bind CsrS. As described above, expression of RocA_TMD_ was also sufficient to restore CsrRS-mediated gene regulation (Fig. 1C). Taken together, these results suggest that the TMDs mediate the functional interaction of RocA with CsrS.

Next, we assessed whether the cytoplasmic domain plays a role in the interaction of RocA with CsrS. Due to the localization of this region of the protein in the GAS cytosol, we first performed experiments with strain M1_F to confirm that the interaction observed between full-length RocA and CsrS in purified membranes (Fig. 3B) was reproduced when performed using protoplast lysates. In agreement with data from the solubilized membrane fraction, pulldown of RocA_FLAG_ from the protoplast lysates of M1_F, but not control strain M1(–), resulted in coprecipitation of native CsrS (Fig. S3). Accordingly, we performed additional pulldown experiments with the protoplast lysates from GAS strain CYT_F. Native CsrS protein did not coprecipitate with RocA_CYT_ (Fig. S3), indicating that the cytoplasmic domain of RocA does not directly bind CsrS. In addition, expression of the cytoplasmic domain was not sufficient to restore regulatory control in a RocA-negative strain (Fig. 1C), an observation that strongly implicates the interaction of RocA_TMD_ with CsrS as critical in the transcriptional regulation of GAS, possibly by modulation of CsrS kinase activity and consequent phosphorylation of CsrR.

10.1128/mBio.01495-19.3 FIG S3 The cytoplasmic domain of RocA does not interact with CsrS. Results of pulldown experiments of RocA_CYT_ with CsrS are shown. RocA_FLAG_ and the cytoplasmic portion of RocA were expressed from plasmid pDL278 in GAS M18 and immunoprecipitated from GAS protoplast lysates using anti-FLAG beads (lanes L, lysate; lanes E, eluted fraction). Fractions were visualized by SDS-PAGE, and immunoblots were probed with anti-CsrS (A) or anti-FLAG (B). The eluted fraction of RocA_CYT_ showed enrichment for RocA but not coprecipitation of native CsrS compared with full-length RocA (M1_F). See the legend of Fig. 1 for strain designations. Download FIG S3, PDF file, 0.4 MB. Copyright © 2019 Lynskey et al. 2019 Lynskey et al. This content is distributed under the terms of the Creative Commons Attribution 4.0 International license.

### The RocA cytoplasmic domain forms homodimers.

Having shown a requirement for the TMDs in the binding of RocA to CsrS, we sought to ascertain a role for the cytoplasmic domain. Although predicted to function as a histidine kinase, RocA lacks certain structural motifs typically associated with kinase activity, and evidence of enzymatic activity has not been reported for RocA (22). In addition, since RocA requires functional CsrS in order to exert its regulatory influence (23) and we have shown the interaction with CsrS to be essential for activity, it seemed likely that the cytoplasmic domain plays an important, nonenzymatic role in RocA function. It is also important to note that while a multicopy plasmid encoding RocA_M3_ (or RocA_TMD_) was sufficient to restore regulatory control to a RocA-negative strain, RocA_M3_ produced from a single chromosomal copy of *rocA*_M3_, as carried by all M3 isolates sequenced to date, is functionally inactive (23, 28). The fact that this truncated form of RocA is defective in its functional interaction with CsrS raised the question of whether the cytoplasmic domain plays a structural role in RocA’s ability to interact with CsrS. Since many membrane-associated sensor kinases with a cytoplasmic domain structure similar to that of RocA form homodimers, which are essential for protein activity (1, 16, 17), we investigated whether RocA could form homodimers and if the cytoplasmic domain was involved in the interaction.

**FIG 4:**
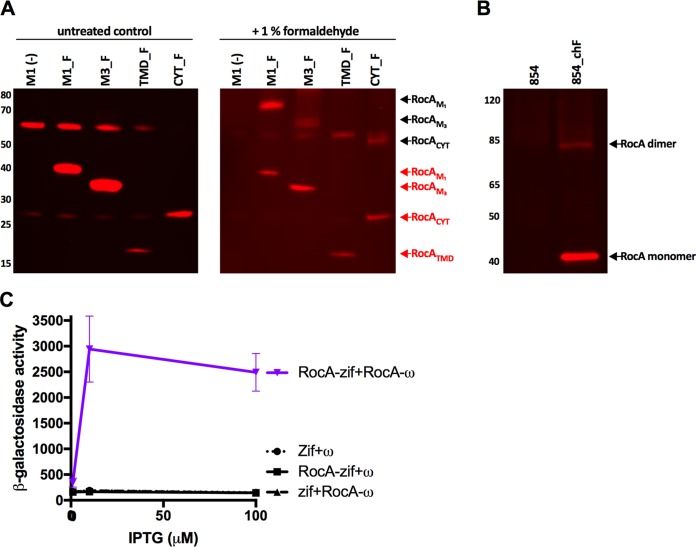
The RocA cytoplasmic domain is necessary for homodimer formation. (A) GAS M18 strains expressing RocA_FLAG_ fragments were subjected to formaldehyde cross-linking, and then RocA was detected in protoplast preparations by SDS-PAGE and immunoblot probed with anti-FLAG antibody (red text = monomer; black text = dimer). See Fig. 1 legend for strain designations. (B) Pull-down of RocA_FLAG_ from the protoplast lysate of GAS M1 strain 854 or 854_chF (854 expressing a single chromosomally integrated copy of RocA_FLAG_). RocA forms homodimers at physiological concentrations within GAS. (C) A bacterial two-hybrid assay demonstrates the interaction between RocA_CYT_ monomers. Contact between RocA_CYT_ fused to the ω subunit of E. coli RNA polymerase and RocA_CYT_ fused to the V-Zif protein results in transcription activation from the *lac* operon quantifiable by β-galactosidase activity (data show means ± standard deviations [SD] of results from 3 independent experiments).

To determine whether full-length RocA could form homodimers in GAS, we treated strains M1(–) and M1_F with formaldehyde, which forms covalent linkages between proteins within 2 Å of one another. After formaldehyde cross-linking, proteins from protoplast preparations of these bacteria were separated by SDS-PAGE, subjected to Western blotting with anti-FLAG antibody to detect RocA, and analyzed for the appearance of a higher-molecular-weight band corresponding to RocA dimers. A new dominant band, absent from strain M1(–) and approximately twice the size of a RocA monomer, was detected in the protoplast lysate of strain M1_F, a result that suggests strongly that homodimer formation occurs within GAS (Fig. 4A). A less abundant higher-molecular-weight complex, approximately the predicted size of a RocA tetramer, was also observed. We also generated a strain of serotype M1 GAS, designated 854_chF, that contains a single functional chromosomal copy of RocA_FLAG_ (Table 1). Similar cross-linking experiments were performed using a protoplast lysate from this strain. Due to the low native expression levels of RocA, RocA_FLAG_ was affinity purified from protoplast lysates of 854_chF to allow detection by immunoblotting (Fig. 4B). These experiments showed that, at native expression levels, RocA forms homodimers within GAS, suggesting that homodimerization of RocA occurs under physiologic conditions (Fig. 4B).

We also investigated which domains of RocA were necessary for homodimer formation. Under the same cross-linking conditions described above, RocA fragments from the protoplast preparations of strains M3_F and CYT_F were also observed to form homodimers (Fig. 4A). RocA dimers were not observed in TMD_F, which expresses only the N-terminal portion of RocA containing the transmembrane domains. Together, results of these experiments indicate that only the cytoplasmic domain is involved in RocA dimer formation (Fig. 4A). Interestingly, the extent of homodimer formation observed for RocA_M3_ appeared less than that of RocA_M1_, suggesting that dimer formation may be impaired in serotype M3 GAS, which lacks the C-terminal 35 amino acids of the cytoplasmic domain (Fig. 4A).

To further validate that the cytoplasmic domain of RocA is sufficient for RocA-RocA interactions, we utilized a bacterial two-hybrid system. The cytoplasmic domain of RocA_M1_ was cloned into two expression vectors as a C-terminal fusion to either the ω subunit of E. coli RNA polymerase or the V-Zif transcription factor (31). When coexpressed in an E. coli reporter strain, interaction between RocA fragments activates transcription from a test promoter containing a V-Zif-binding site. The test promoter is positioned to drive expression of a linked *lacZ* reporter gene, which can be quantified as β-galactosidase activity (31). As anticipated from the cross-linking experiments, data from these experiments supported the finding that the RocA cytoplasmic domain forms homodimers. Taken together, the results suggest that the cytoplasmic domain is both necessary and sufficient for the formation of RocA homodimers *in vivo* and likely plays a role in RocA function (Fig. 4C).

### RocA_M3_ forms aggregates *in vivo*, which may explain the loss of protein function.

**FIG 5:**
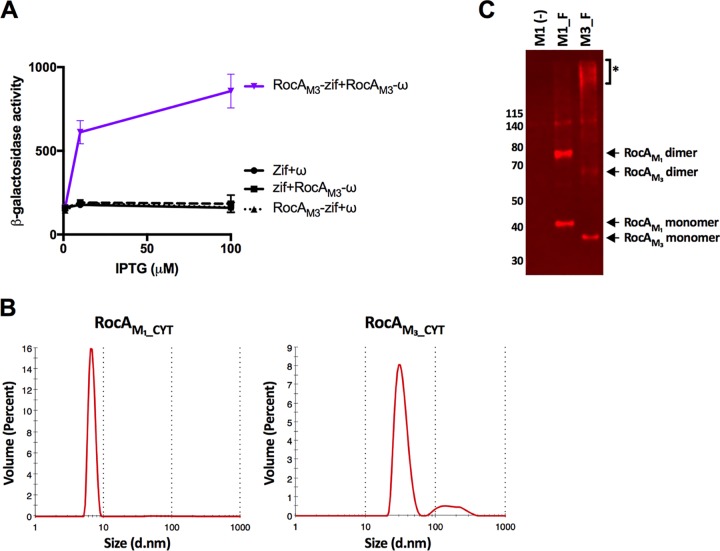
Serotype M3 RocA protein aggregates *in vitro* and *in vivo*. (A) A bacterial two-hybrid assay demonstrates the interaction between RocA_M3_CYT_ monomers. Contact between RocA_M3_CYT_ fused to the ω subunit of E. coli RNA polymerase and RocA_M3_CYT_ fused to the V-Zif protein results in transcription activation from a test promoter driving the expression of a linked *lacZ* reporter gene, quantifiable by β-galactosidase activity (data show means ± SD of results from 3 independent experiments). (B) Dynamic light-scattering analysis showing relative molecular sizes of recombinant RocA_M1_CYT_ and RocA_M3_CYT_ proteins. RocA_M1_CYT_ eluted from a Ni-NTA resin column as a soluble monomer of the expected molecular size, whereas RocA_M3_CYT_ formed large aggregates. d.nm, diameter in nanometers. (C) Protoplast preparations of a GAS M18 strain expressing FLAG-tagged RocA_M1_ or RocA_M3_ after formaldehyde cross-linking. Samples were subjected to SDS-PAGE, and the immunoblot was probed with anti-FLAG antibody. *, high-molecular-weight aggregates of RocA_M3_, but not RocA_M1_. See the legend of Fig. 1 for strain designations.

Having observed that the cytoplasmic domain of RocA is necessary for functional interaction of RocA with CsrS and that homodimer formation may be less efficient in M3 GAS strains, which express a truncated form of RocA (Fig. 4A), we hypothesized that impaired dimerization contributes to the loss of RocA activity observed in M3 GAS strains. However, bacterial two-hybrid assays using RocA_M3-CYT_ similar to those described above revealed that the cytoplasmic domain of RocA_M3_ was capable of interacting with itself (Fig. 5A), albeit less avidly than the (full-length) cytoplasmic domain of RocA_M1_ (Fig. 4C).

To compare the relative affinities of RocA_M1_ and RocA_M3_ homodimer interactions, we purified recombinant proteins corresponding to the cytoplasmic domains of the two RocA proteins. We first expressed RocA_M1_, which could be purified at high concentration and which remained soluble in aqueous buffers (Fig. 5B). However, under the same conditions and at a comparable concentration, RocA_M3_ formed high-molecular-weight soluble aggregates, as assessed by dynamic light scattering (Fig. 5B). Aggregation of RocA_M3_ prevented a direct analysis of the affinity of binding *in vitro*.

In order to ascertain whether RocA_M3_ forms aggregates *in vivo* within M3 GAS, we performed additional cross-linking experiments to detect such aggregates in the bacterial cells. SDS-PAGE and Western blotting of protoplast preparations prepared from GAS cells after formaldehyde cross-linking revealed high-molecular-weight aggregates of RocA_M3-FLAG_ in strain M3_F but not in strain M1_F (expressing full-length RocA_M1-FLAG_) (Fig. 5C). These results are consistent with the protein aggregation observed *in vitro* for the recombinantly expressed cytoplasmic domain of RocA_M3_ compared to that of RocA_M1_ (Fig. 5B). We speculate that aggregation of RocA_M3_ may explain the defective regulation of the CsrRS regulon in M3 isolates, presumably by impairing the functional interaction of RocA_M3_ with CsrS.

## DISCUSSION

In this study, we demonstrate that RocA is an auxiliary regulator of the CsrRS TCS in GAS and that RocA and CsrRS collectively form a multicomponent integrated regulatory system. Using a combination of genetic and biochemical approaches, we show that the membrane-associated N-terminal region of RocA interacts with CsrS in the bacterial membrane and is essential for functional regulation of the CsrRS regulon. In addition, we show that the RocA cytoplasmic domain is necessary for the formation of homodimers, a process suggested to be an important step in RocA’s modulation of CsrRS function. Finally, we demonstrate that in M3 GAS, truncated RocA_M3_ forms aggregates, a process associated with impairment of a functional interaction with CsrS.

Auxiliary regulators are increasingly recognized for the significant role that they play in bacterial gene regulation (16–18). These proteins are distinct from the TCS on which they act but through direct protein-protein interactions are capable of modulating the activity of either the sensor kinase (within the sensing, intramembrane, or enzymatic domains) or the response regulator to ultimately control its phosphorylation state and regulatory activity (19). Here, we demonstrate binding of RocA to CsrS *in vivo* and identify the RocA domains required for functional interaction with the CsrRS TCS.

CsrS is a bifunctional sensor capable of modulating CsrR phosphorylation by both kinase and phosphatase activity (15, 32). Current models suggest that sensor histidine kinases dimerize to permit one kinase molecule to phosphorylate the other (33). Conformational changes in kinase homodimers in response to external ligands or regulatory proteins mediate autophosphorylation and subsequent phosphotransfer to the response regulator to modulate its binding to target promoters (33). CsrS is known to modulate expression of the CsrRS regulon in response to increased extracellular Mg^2+^ (6) and subinhibitory levels of the cathelicidin LL-37 (11), inducing repression and derepression, respectively, of most target genes. Here we show that the region of RocA that includes TMDs interacts with CsrS in the GAS membrane, and we hypothesize that the consequence of this interaction is to modulate the conformation of CsrS and its subsequent phosphorylation or dephosphorylation of CsrR. RocA enhances transcriptional repression of target genes by increasing the abundance of phosphorylated CsrR, suggesting that RocA interactions with CsrS enhance its kinase activity or inhibit its phosphatase activity (23, 24).

Deletion of *rocA* has been shown to impair the GAS response to LL-37 (27). While it is possible that RocA is actively involved in detecting extracellular LL-37 levels, since RocA has no extracellular sensing domain, it seems more likely that, in the absence of RocA, CsrS kinase activity is tonically inhibited or that its phosphatase activity is enhanced, mimicking the LL-37 response (14). LL-37 binding to CsrS may counteract the effect of RocA, allowing the bacteria to respond rapidly to changing environments. In support of this hypothesis is the observation that LL-37 regulation of the CsrRS regulon in serotype M3 GAS is less marked than that observed for other serotypes believed to express functional RocA protein (6).

GAS strains isolated from patients with invasive infection are frequently associated with function-altering mutations in CsrS or CsrR (7, 34). However, such mutations are rare among noninvasive throat isolates, and experimental studies suggest that they are deleterious for colonization of the host skin and oropharynx (35). Mutations in RocA have also been observed in clinical disease isolates and are associated with enhanced virulence (25, 36, 37). A recent study showed that the *rocA* gene in serotype M28 GAS is unusually polymorphic, with a significantly higher frequency of mutation than in other serotypes (25). The majority of mutations were identified within the N-terminal TMDs of RocA, which we show are both necessary and sufficient for interactions with CsrS. Serotype-specific mutations resulting in truncated, nonfunctional RocA have also been reported for M3 and M18 GAS (23, 26, 28). Mutations in RocA are therefore not necessarily deleterious to the bacterium and have been shown to underlie increased virulence factor expression and infection syndromes associated with these serotypes.

The serotype M18 truncation in RocA occurs at amino acid 29 of 451 (26). In contrast, the serotype M3 truncation occurs at amino acid 416 of 451, generating a large fragment of RocA protein (28). The RocA_M3_ protein has been shown to be nonfunctional when it is expressed from the GAS chromosome at physiological levels (23). Our finding, supporting a previous report (27), that the RocA TMDs (amino acids 1 to 225) are sufficient for a functional interaction with CsrS raised the question as to how the M3 truncation impacts RocA activity. Subsequent cross-linking experiments wtih GAS and bacterial two-hybrid assays with E. coli revealed that the cytoplasmic domain of RocA is both necessary and sufficient for homodimer formation and that dimerization is likely important for RocA activity at physiological expression levels. Interestingly, the truncated serotype M3 cytoplasmic domain was also able to form homodimers, albeit less efficiently than the full-length cytoplasmic domain of the M1 RocA protein. Subsequent experiments demonstrated that RocA_M3_ forms aggregates within GAS, which we hypothesize contributes to the loss of function of RocA in serotype M3 GAS, due to impaired homodimer formation and the loss of a functional interaction with CsrS.

Homologs of RocA have not been described for other bacterial species despite the fact that CsrRS homologs are expressed in a number of other streptococci, the best studied of which is that expressed by the human pathogen group B *Streptococcus* (GBS) (38, 39). Interestingly, Abx1, a membrane protein with eight TMDs, has been shown to modulate the activity of CsrS in GBS. In contrast to RocA, Abx1 acts to reduce the abundance of phosphorylated CsrR to derepress virulence factor production (16). Abx1 has also been shown to interact directly with the TMDs of GBS CsrS, suggesting that this may be a common theme in the modulation of CsrS activity (16). Abx1 is also capable of forming homodimers; however, the effect of dimerization on gene regulation has not been reported. The variation in auxiliary regulators may allow a controlled response to external stimuli within the varied host niches inhabited by different streptococcal species.

In this study, we have demonstrated that RocA acts as an auxiliary regulator of the GAS TCS CsrRS and that RocA and CsrS interact in the bacterial membrane to modulate the phosphorylation state of the response regulator CsrR. The evidence presented supports a direct interaction between an auxiliary regulatory protein and CsrS, the activity of which is also regulated by Mg^2+^ and LL-37. The relative kinase/phosphatase activity of CsrS is thus the result of the integration of environmental signals and input from RocA that together result in a complex regulatory system critical for GAS colonization and infection in the human host.

## MATERIALS AND METHODS

### Bacterial strains and growth conditions.

The strains used for molecular manipulation were GAS 854, an M1 strain isolated from a patient with a retroperitoneal abscess (11), GAS 188, an isogenic acapsular derivative of M3 strain 950771 (40) and H566, an M18 strain isolated from a patient with streptococcal toxic shock syndrome (26). All strains used are listed in Table 1. GAS were cultured on Trypticase soy agar supplemented with 5% defibrinated sheep blood (Remel) or Todd-Hewitt (TH) agar or in TH yeast (THY) broth at 37°C in the presence of 5% CO_2_. E. coli strain NEB5α (New England Biolabs) was used for cloning and BL21(DE3) (New England Biolabs) for recombinant protein expression. E. coli was cultured in LB broth (Sigma). Lactococcus lactis subsp. *cremoris* 1363 was grown at 30°C in GM17 broth or on GM17 agar (41). Growth media were supplemented with antibiotics where appropriate at the following concentrations: for E. coli, spectinomycin at 50 μg/ml, erythromycin at 500 μg/ml, kanamycin at 30 μg/ml, and tetracycline at 10 μg/ml; for GAS, spectinomycin at 100 μg/ml and erythromycin at 1 μg/ml; and for L. lactis, erythromycin at 5 μg/ml.

### Generation of GAS strains expressing RocA fragments.

The *rocA*_M1_ sequence was amplified by PCR from genomic DNA extracted from strain 854 and cloned into the vector pDL278 as described previously (26). A FLAG tag was incorporated immediately upstream of the *rocA* stop codon by overlap PCR using primers pDLrocA_FLAG__F and _R (Table 2), which incorporate XmaJI sites into both ends of the PCR product. The resulting product was digested with XmaJI and DpnI (Thermo Scientific) to cleave residual methylated template plasmid and ligated using T4 ligase (Thermo Scientific). The resulting construct was designated pDL_rocA_M1FLAG_.

**TABLE 2:** Primers used in this study

Primer name	Sequence 5′–3′^*a*^
pDLrocA_FLAG__F	GGC**CCTAGG**TGATGTTAAAGGTATGAA
pDLrocA_FLAG__R	GGC**CCTAGG**CTTGTCGTCATCGTCTTTGTAGTCGTCAGGCTTAGCTATTTC
pDL_rocA_M3__F	CG**GGATCC**TTGCAAAAACTGTAGGCTGTG
pDL_rocA_M3__R	CG**GGATCC**TTACTTGTCGTCATCGTCTTTGTAGTCAGTCAAATTGCCAGTC
pDL_rocA_TMD_FLAG__F	GGC**CCTAGG**TGAAACTCTATTGAGGCAATTG
pDL_rocA_TMD_FLAG__R	GGC**CCTAGG**CTTGTCGTCATCGTCTTTGTAGTCTTGTTTTACATAACGCTC
pDL_rocA_CYT_FLAG__F	GGAATTC**CATATG**AACTCTATTGAGGCAATTGTGC
pDL_rocA_CYT_FLAG__R	GGAATTC**CATATG**TTATCCTTCTCCTTCTCGTTC
chFLAG_1F	CG**GGATCC**GAATGCTGAAAAGAATAATGC
chFLAG_1R	TCACCTAGGCTTGTCGTCATCG
chFLAG_2F	GATGACGACAAGCCTAGGTGATGTTAAAGGTATGAA
chFLAG_2R	CGCC**GTCGAC**CCCAACACCTGAAGCCCAGTG
L_csrS_F	CG**GGATCC**GAAAATCGAGATGTGATAA
L_csrS_R	CTCACGAATAACGTATCACCTAGGCTTGTCGTCATC
L_rocA_F	GACAAGCCTAGGTGATACGTTATTCGTGAGAAATA
L_rocA_R	CTAATGATGATGATGATGATGCTCTCTTTAGACTGGGCC
L_rocA_R_PstI	CGCC**CTGCAG**CTAATGATGATGATGATG
pET_ATPase_NcoI_F	CATG**CCATGG**AAAAGAATAATGCTAAAGATG
M1_pET_ATPase_HIS_R	CG**GGATCC**TCAATGATGATGATGATGATGGTCAGGCTTAGCTATTTC
M3_pET_ATPase_HIS_R	CG**GGATCC**TCAATGATGATGATGATGATGAGTCAAATTGCCAGTCATC
RocA2H_F	AAGGAAAAAA**GCGGCCGC**AAACTCTATTGAGGCAATTGTGC
RocA2H_R	CG**GGATCC**TCAGTCAGGCTTAGCTATTTC
M3RocA2H_R	CG**GGATCC**TTAAGTCAAATTGCCAGTCAT
CsrS T284A F	CATGAATTACGAGCGCCGGTTGCG
CsrS T284A R	CGCAACCGGCGCTCGTAATTCATG
BamHI-csrS-5'	CCCC**GGATCC**ATGGAAAATCAGAAACAAAAACAG
csrS-ID-3'-SalI	CCCC**GTCGAC**TTACTAACTCTCTTTAGACTGGGC

a Bold with underlining indicates restriction endonuclease sites.

The *rocA*_M3_ sequence was amplified from genomic DNA extracted from strain 188 using primers pDL_rocA_M3__F and _R (Table 2) and cloned into the vector pDL278 using restriction enzyme BamHI (Thermo Scientific) and T4 ligase (Thermo Scientific). The resulting construct was designated pDL_rocA_M3FLAG_.

Plasmid pDL_rocA_TMD_FLAG_, expressing the N-terminal RocA TMDs, was generated from plasmid pDLrocA_M1_FLAG_ by overlap PCR. The entire plasmid was amplified using primers pDL_rocA_TMD_FLAG__F and _R (Table 2) to incorporate a stop codon at amino acid 226 and XmaJI restriction sites at both ends of the amplicon. The resulting PCR product was digested with XmaJI and DpnI (Thermo Scientific) to cleave residual methylated template plasmid and ligated using T4 ligase (Thermo Scientific).

Plasmid pDL_rocA_CYT_FLAG_, expressing the C-terminal RocA cytoplasmic domain was generated from plasmid pDLrocA_M1_FLAG_ by overlap PCR. Primers pDL_rocA_CYT_FLAG__F and _R (Table 2) were used to amplify pDLrocA_M1_FLAG_ and remove amino acids 1 to 225 of the *rocA* gene product from the resulting PCR product. NdeI restriction sites were incorporated into both ends of the amplicon, providing a new, in-frame start codon for expression of RocA_CYT_FLAG_ *in vivo*. The resulting PCR product was digested with NdeI and DpnI (Thermo Scientific) to cleave residual methylated template plasmid and ligated using T4 ligase (Thermo Scientific).

All resulting plasmids were transformed into GAS strain H566 by electroporation as described previously (26).

### Allelic-exchange mutagenesis.

Using the temperature-sensitive E. coli-GAS shuttle vector pJRS233 (42), plasmid pJRS_rocA_FLAG_ was generated to facilitate creation of an isogenic GAS-M1 strain expressing a single, native chromosomal copy of *rocA* with a FLAG tag. Primers chFLAG_1F and _R (Table 2) were used to amplify *rocA* from genomic DNA purified from M1 strain 854 and to incorporate a C-terminal FLAG tag into the PCR product (sequence, GACTACAAAGACGATGACGACAAG) directly upstream of the native stop codon, generating amplicon ch1. Primers chFLAG_2F and _R (Table 2) were used to amplify a second PCR product (ch2) that overlaps ch1. Splicing by overlap extension (SOEing) PCR, using overlapping amplicons ch1 and ch2 as target DNA, was performed using primers chFLAG_1F and chFLAG_2R (Table 2), incorporating the restriction sites BamHI and SalI into the resulting PCR product to facilitate cloning into the vector pJRS233. The resulting shuttle vector pJRS_rocA_FLAG_ was transformed into GAS-M1 strain 854 by electroporation (26), and *rocA*_FLAG_ was exchanged with the chromosomal copy of *rocA* by allelic-exchange mutagenesis (43). Sanger sequencing was performed to confirm introduction of the FLAG tag into the chromosomal *rocA*_M1_ gene.

pJRS233 was also used to generate an isogenic GAS-M1 strain expressing a single, native chromosomal copy of *csrS* with a single mutation at nucleotide 852 (A to G), resulting in a nonsynonymous mutation from threonine to alanine at amino acid 284. Plasmid pJRS_csrS_T284A_ was generated by SOEing PCR. Overlapping amplicons were generated from genomic DNA purified from M1 strain 854 using primer pairs BamHI-csrS-5′/CsrS T284A R and CsrS T284A F/csrS-ID-3′-SalI (Table 2) to incorporate the A852G nucleotide change into the *csrS* gene. SOEing PCR, using the overlapping amplicons as target DNA, was performed using primers BamHI-csrS-5′ and csrS-ID-3′-SalI (Table 2), incorporating the restriction sites BamHI and SalI into the resulting PCR product to facilitate cloning into the vector pJRS233. The resulting plasmid, pJRS_csrS_H284A_, was transformed into GAS-M1 strain 854 by electroporation (26). *csrS*_H284A_ was exchanged with the chromosomal copy of *csrS* by allelic-exchange mutagenesis as described previously (43) and confirmed by Sanger sequencing.

### Generation of L. lactis coexpressing CsrS_His_ and RocA_FLAG_.

A DNA fragment containing *csrS*_His_ and *rocA*_FLAG_ was generated by SOEing PCR and cloned into the vector pOri23, an E. coli Gram-positive shuttle vector (41) provided by June Scott (44). Briefly, primers L_csrS_F + R and L_rocA_F + R (Table 2) were used to amplify overlapping PCR products containing *csrS*_6×His_ and *rocA*_FLAG_. Using these two amplicons as target DNA, SOEing PCR was performed with primers L_csrS_F and L_rocA_R_PstI (Table 2) to generate a single amplicon containing *csrS*_6×His_ and *rocA*_FLAG_ and incorporate BamHI and PstI restriction sites. The resulting amplicon was then cloned into the vector pOri23 to generate pOri23_*csrS*_His_*rocA*_FLAG_ and transformed into L. lactis as described previously (45).

### Recombinant RocA expression.

The predicted cytoplasmic domains of RocA_M1_ and RocA_M3_ were amplified from strain 854 and 188 genomic DNA using primer pET_ATPase_NcoI_F with reverse primers M1_pET_ATPase_HIS_R and M3_pET_ATPase_HIS_R, respectively (Table 2). The resulting PCR products were cloned into the vector pET19b (Novagen), facilitated by incorporation of NcoI and BamHI restriction sites. Restriction digestion with NcoI and BamHI resulted in removal of the N-terminal His tag included within the plasmid. The reverse primers incorporated a C-terminal His tag within recombinant RocA. The resulting constructs, designated pET19b_RocA_M1_ and pET19b_RocA_M3_, were transformed into BL21 cells for recombinant protein expression.

Transformants were initially cultured to an *A*_600_ of 0.3 at 37°C and then to an *A*_600_ of 0.5 at 20°C. Protein expression was induced at 20°C using 0.5 mM isopropyl-β-D-thiogalactopyranoside (IPTG) for 3 h. Cells were harvested by centrifugation, lysed in lysis buffer [20 mM Tris, 200 mM NaCl, 10% glycerol, 1 mM Tris(2-carboxyethyl)phosphine hydrochloride (TCEP), 20 mM imidazole] supplemented with protease inhibitor cocktail (GE Healthcare) lysozyme (1 mg/ml) and DNase I (0.05 mg/ml) and subjected to sonication (Sonic Dismembrator; Thermo Scientific). Lysates were filtered with a 0.2-μm-pore-size filter and purified over Ni-NTA resin (1-ml resin bed). The resin was washed with 20 column volumes of lysis buffer without protease inhibitors and eluted with 350 mM imidazole in the same buffer. Purified proteins were subjected to dynamic light scattering analysis (Malvern).

### Capsule quantification.

GAS strains were cultured in Todd-Hewitt broth (THB) and capsule quantified as described previously (26) using the hyaluronan DuoSet (R&D).

### Preparation of bacterial protoplasts and cell fractions. (i) Preparation of protoplasts.

GAS (180 ml/strain) or L. lactis (90 ml/strain) were cultured to an *A*_600_ of 0.4 and pelleted, and cell walls were digested in 44 ml 10 mM Tris supplemented with 30% raffinose, mutanolysin (100 units/ml) (Sigma), lysozyme (1 mg/ml) and protease inhibitor cocktail (1:100) (Calbiochem) for 1 h at 37°C. Samples were centrifuged at 4°C, and pellets containing protoplasts were harvested and stored at −20°C.

**(ii) Preparation of protoplast lysates.** Frozen protoplasts were resuspended in 1 ml TBS_150_ (50 mM Tris, 150 mM NaCl, pH 7.4) and subjected to sonication (Bioruptor) for 5 cycles (5 min; 30 s on, 30 s off). Cellular debris was removed by centrifugation (20,000 × *g*, 30 min, 4°C). The resulting supernatants were either incubated in 1-ml aliquots with anti-FLAG magnetic beads (Sigma) (50 μl beads/1 ml lysate) at 4°C overnight, with rotation, or filtered with a 0.2-μm-pore-size filter and further processed for membrane preparation.

**(iii) Preparation of membrane and cytosolic fractions.** Membranes were pelleted by ultracentrifugation (150,000 × *g*, 2 h, 4°C). Supernatants constituted the cytosolic fraction. Membrane pellets were homogenized in ice-cold TBS_150_ and solubilized in 1% *n*-dodecyl-β-d-maltoside (DDM) on ice (1 h). Samples were diluted 1:4 in ice-cold TBS_150_ and incubated in 1-ml aliquots with anti-FLAG magnetic beads (Sigma) (50 μl beads/1 ml membrane preparation) overnight (4°C, with rotation).

### Immunoprecipitation of RocA_FLAG_.

Following overnight incubation, RocA_FLAG_ was immunoprecipitated from cytoplasmic and membrane preparations according to the manufacturer’s instructions, with some minor modifications. Anti-FLAG beads were washed 3 times (4°C, 15 min with rotation in TBS_500_ [50 mM Tris, 500 mM NaCl, pH 7.4]), supplemented with 0.1% NP-40 (for protoplast preparations) or 0.1% DDM (for membrane preparations), and RocA_FLAG_ eluted with 3× FLAG in the same buffers. Eluted fractions were concentrated 20× using 10-kDa spin columns (Thermo Scientific) and subjected to SDS-PAGE and Western blotting.

### Affinity purification of CsrS_His_.

Membrane preparations were purified over Ni-NTA resin (1-ml resin bed). The resin was washed with 2.5-ml column volumes of lysis buffer supplemented with 0.1% DDM, centrifuged (100 × *g*, 2 min, 4°C) to remove any unbound protein, and resuspended in 5 ml of the same buffer. Resin was allowed to settle and washed with 10 column volumes of lysis buffer. Bound CsrS_His_ was eluted with 500 mM imidazole in the same buffer. Eluted fractions were concentrated 10× using 10-kDa spin columns (Thermo Scientific) and subjected to SDS-PAGE and Western blotting.

### Cross-linking of GAS proteins *in vivo*.

GAS strains were cultured to an *A*_600_ of 0.4 and incubated with 1% formaldehyde (10 min, room temperature, with rotation) prior to addition of 250 mM glycine to quench residual formaldehyde (20 min, room temperature, with rotation). Pellets were washed twice in phosphate-buffered saline (PBS) and protoplast preparations purified as described above. Samples were subjected to SDS-PAGE and Western blotting.

### SDS-PAGE and Western blotting.

For SDS-PAGE, samples were denatured with 100 mM dithiothreitol (DTT) and 1× Laemmli buffer. Proteins were fractionated on either 4 to 12% NuPAGE Novex Bis-Tris gels or 7% NuPAGE Tris-acetate gels (Thermo Scientific). Proteins were transferred onto nitrocellulose membranes (Thermo Scientific), blocked (5% skim milk powder; Sigma), and probed with anti-FLAG (Sigma), anti-CsrS (6, 12), or anti-His (Abcam) antibodies. Proteins were visualized and analyzed using an Odyssey infrared imaging system (LI-COR, Lincoln, NE) and Image Studio software monitoring incubation with fluorescent secondary antibodies (Thermo Scientific).

### Detection of phosphorylated CsrR protein in GAS.

GAS strains were cultured to an *A*_600_ of 0.5 in 10 ml THY broth supplemented with 30 μg/ml hyaluronidase and harvested by centrifugation. Bacterial lysates were prepared rapidly by bead beating them (twice for 16 s) in ice-cold PBS. All samples were kept chilled to minimize spontaneous dephosphorylation. Dephosphorylated control samples were prepared by heating them to 95°C for 10 min. Samples were fractionated by SDS-PAGE on 10% acrylamide gels supplemented with Phos-tag reagent (Wako) in electrode buffer (25 mM Tris, 192 mM glycine, 0.1% SDS) at 150 V for 2 h. Gels were washed in transfer buffer (Thermo Scientific) twice but first supplemented with 1 mM EDTA. Western blotting was then performed as described above, and blots were probed with anti-CsrR antiserum (12). Relative abundances of CsrR protein were quantified using Image Studio software.

### Bacterial two-hybrid assays.

The C-terminal fragments of *rocA*_M1_ and *rocA*_M3_ gene products were amplified by PCR with primer RocA2H_F paired with RocA2H_R and M3RocA2H_R, respectively. The restriction sites NotI and BamHI were incorporated to facilitate cloning in-frame into the vectors pACTR-ω-GP and pBR-V-Zif-AP as described previously (31). The resulting vectors were transformed into E. coli strain KDZif1ΔZ, and protein interactions were assessed by β-galactosidase assay (46).
