# Supplementary material for: RocA Binds CsrS To Modulate CsrRS-Mediated Gene Regulation in Group A Streptococcus
Source: mBio. 2019 Jul 16;10(4):e01495-19. doi: 10.1128/mBio.01495-19 (PMC6635533; doi:10.1128/mBio.01495-19)
Supplement: FIG S1 [file mBio.01495-19-sf001.pdf]

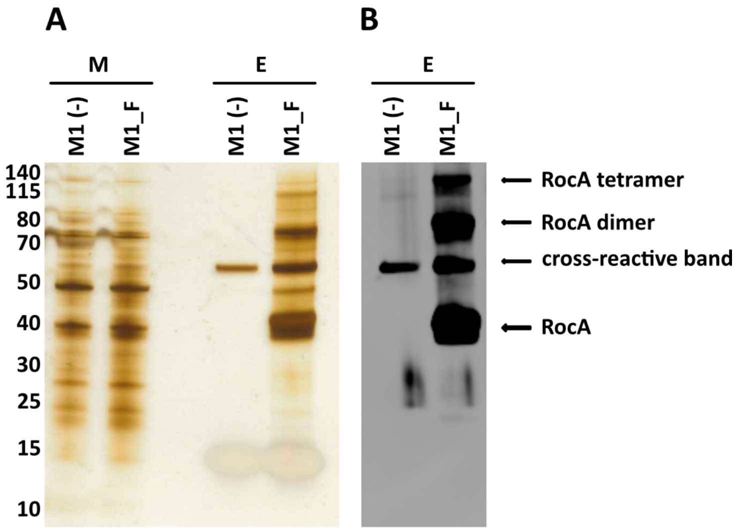

Figure S1: Immunoprecipitation of RocA$_{FLAG}$ from a membrane preparation of GAS strain M1_F pulls down CsrS and only minimal amounts of other proteins. Panel A shows a silver-stained SDS-PAGE gel of the membrane preparations (M) and eluted fractions (E) from the anti-FLAG affinity resin used in the experiments shown in Figure 2. The strains tested are M1(-) and M1_F, expressing RocA without and with a FLAG-tag, respectively. Panel B is an anti-FLAG western blot run on the same samples. The only band observed in the eluted fraction of M1(-) is the nonspecific band representing a protein expressed by GAS that cross-reacts with the anti-FLAG antibody. All the major protein bands observed in the eluted fraction of strain M1_F by silver stain are observed in the anti-FLAG western blot, suggesting that these bands were specifically pulled down by immunoprecipitation. Many protein bands visible in the membrane preparation of M1(-) by silver stain are not visible in the eluted fraction, in particular, the proteins smaller than 40 kDa. These results suggest that the pull-down is largely free of unrelated proteins, and the co-elution of CsrS with RocA$_{FLAG}$ reflects a specific interaction.
