# Supplementary material for: RocA Binds CsrS To Modulate CsrRS-Mediated Gene Regulation in Group A Streptococcus
Source: mBio. 2019 Jul 16;10(4):e01495-19. doi: 10.1128/mBio.01495-19 (PMC6635533; doi:10.1128/mBio.01495-19)
Supplement: FIG S2 [file mBio.01495-19-sf002.pdf]

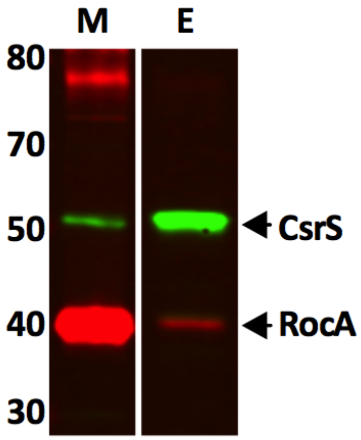

Figure S2: RocA interacts directly with CsrS in the cell membrane of a heterologous bacterial species. $RocA_{FLAG}$ and $CsrS_{his}$ were co-expressed from plasmid pOri23 in *L. lactis*. Bacterial membranes (M) were isolated, solubilized, and the proteins were subjected to affinity purification using Ni-NTA resin. SDS-PAGE and immunoblot of the fraction eluted from the resin (E) showed enrichment for $CsrS_{his}$ and co-precipitation of $RocA_{FLAG}$ (red=anti-FLAG, green=anti-his).
