# Supplementary material for: RocA Binds CsrS To Modulate CsrRS-Mediated Gene Regulation in Group A Streptococcus
Source: mBio. 2019 Jul 16;10(4):e01495-19. doi: 10.1128/mBio.01495-19 (PMC6635533; doi:10.1128/mBio.01495-19)
Supplement: FIG S3 [file mBio.01495-19-sf003.pdf]

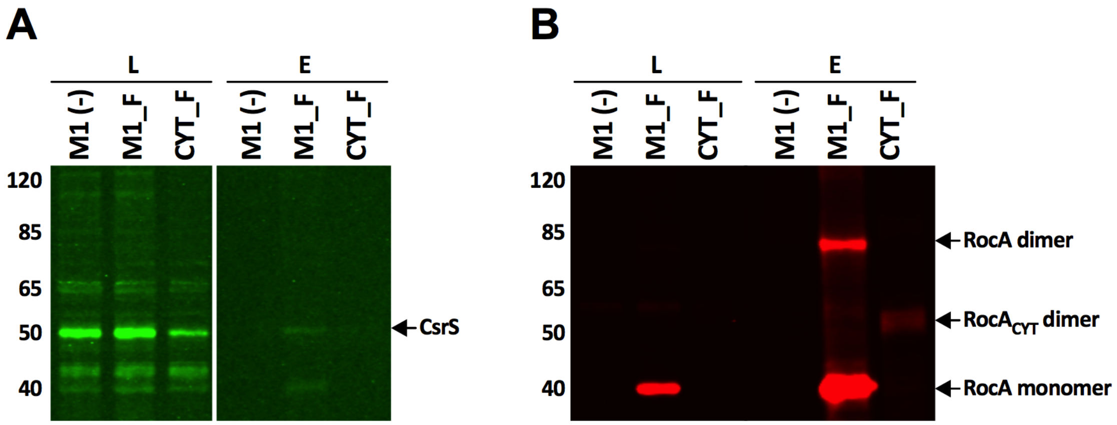

Figure S3: Cytoplasmic domain of RocA does not interact with CsrS. Pull-down experiments of RocA$_{CYT}$ with CsrS. RocA$_{FLAG}$ and the cytoplasmic portion of RocA were expressed from plasmid pDL278 in GAS M18 and immunoprecipitated from GAS protoplast lysates using anti-FLAG beads (L = lysate, E = eluted fraction). Fractions were visualized by SDS-PAGE and immunoblot probed with anti-CsrS (A) or anti-FLAG (B). Eluted fraction of RocA$_{CYT}$ showed enrichment for RocA, but not co-precipitation of native CsrS when compared with full-length RocA (M1_F). See Figure 1 legend for strain designations.
